# Efficacy and safety of traditional Chinese medicine injection in the treatment of ventricular remodeling after acute myocardial infarction: A network meta-analysis

**DOI:** 10.1097/MD.0000000000047584

**Published:** 2026-02-06

**Authors:** Jianlong Xiong, Huan Wu, Tao Xu

**Affiliations:** a The Second Clinical Medical College, Guizhou University of Traditional Chinese Medicine, Guiyang, Guizhou, China; b The Second Affiliated Hospital of Guizhou University of Chinese Medicine, Guiyang, Guizhou, China.

**Keywords:** acute myocardial infarction, clinical efficacy, herbal injection, safety, seticulated meta-analysis, ventricular remodeling

## Abstract

**Background:**

This study attempted to assess the efficacy and safety of different herbal injections in combination with conventional therapy (CT) for the treatment of postinfarction ventricular remodeling using a net meta-analysis.

**Methods:**

The search was conducted as of April 2024 in the China Knowledge Network Database, Chongqing Vip Database (VIP), Wanfang Platform Database (Wanfang), China Biomedical Literature Service System (SinoMed), PubMed, Web of Science Database, EMBASE, Cochrane Library for information about We searched the Cochrane Library for randomized controlled trials (RCTs) of herbal injections for postinfarction ventricular remodeling. Quality was assessed by the Cochrane Risk of Bias tool, and a net meta-analysis was performed to compare the efficacy and safety of different herbal injections in combination with conventional therapies. Data were analyzed and plotted using RevMan5.4, Stata18.0 MP software software.

**Results:**

A total of 1948 records were retrieved, and 24 eligible RCTS were included, involving 2343 patients and 12 injections. The results of network meta-analysis showed that compared with conventional treatment, Shuxuetong injection + CT was significantly more likely to improve the clinical efficacy; Salvianolate injection + CT had the lowest incidence of adverse drug reaction. In terms of improving left ventricular ejection fraction, Guanxin Ning injection + CT was the best; In reducing left ventricular end-diastolic diameter, Xinmailong injection + CT has the best effect; In terms of reducing LVESD, Shenxiong injection + CT is the best. Chuanxiong Rhizoma injection + CT has the best effect on reducing left ventricular end-diastolic volume; In terms of reducing left ventricular end-systolic volume, Shenfu injection + CT has the best effect. The differences among the groups were statistically significant. Fourteen RCTS included reported the occurrence of adverse reactions, and the overall adverse reactions of TCM injection combined with conventional treatment were less than those of the control group.

**Conclusion:**

Current evidence suggests that the use of Chinese herbal injections in addition to conventional therapies may safely and effectively improve ventricular remodeling in patients with acute myocardial infarction and, to varying degrees, enhance cardiac function, quality of life, and prognosis.

## 1. Introduction

Acute myocardial infarction (AMI) refers to the formation of atherosclerosis in the coronary arteries of the heart due to plaque dislodgement, arterial vascular occlusion leading to an acute and sustained reduction or even cessation of coronary blood supply, triggering necrosis of myocardial tissue and impaired function within a short period of time, which is a clinically common cardiovascular critical illness that seriously jeopardizes human health. It is a common clinical cardiovascular critical condition that seriously jeopardizes human health.^[1]^ Ventricular remodeling is the most common form of ventricular remodeling. Ventricular remodeling refers to the slow structural changes that occur within the ventricles under cardiac stress, such as changes in cardiac size, morphology, and function, and is the pathological basis for the occurrence of a variety of adverse cardiovascular events.^[2]^Patients are often prone to clinical events such as heart failure, arrhythmia, and even death after AMI, which have been confirmed by relevant studies to be closely related to ventricular remodeling after MMI.^[3]^ Therefore, it is important to find an effective and safe method to delay or inhibit ventricular remodeling to improve the prognosis of AMI. Chinese medicine, as part of the traditional cultural treasures of China, has accumulated a great deal of experience in the treatment of cardiac diseases, and several studies have confirmed that Chinese medicines have unique advantages in reversing the process of ventricular remodeling and improving cardiac function. As a product of combining modern technology and traditional Chinese medicine, Chinese medicine injection has been widely used in clinical work in recent years, and as a complementary means to conventional treatment, its features such as obvious efficacy and fewer side effects have been recognized by more and more researchers. However, due to the wide variety of TCM injections available on the market and the lack of direct comparisons between injections, there is still no clear conclusion as to which class of TCM injections is relatively optimal in improving the degree of ventricular remodeling and safety after AMI in clinical practice. Therefore, this article uses reticulated meta-analysis to analyze and evaluate the comprehensive comparative ranking of different types of Chinese medicine injections, with the aim of providing evidence-based support for clinical drug selection and use.

## 2. Data and methods

This study mainly followed PRISMA-NMA for reticulated Meta-analysis,^[4]^which has been registered in PROSPERO platform with registration number CRD42024533623.

### 2.1. Inclusion criteria

#### 2.1.1. Types of research

Clinical randomized controlled trials (RCTs), language and type not restricted.

#### 2.1.2. Research objectives

Patients with a clear diagnosis consistent with ventricular remodeling after myocardial infarction, whose diagnostic criteria can be referred to the Expert Consensus on Integrative Diagnosis and Treatment of AMI in Traditional Chinese and Western Medicine,^[5]^ and whose patients’ age, gender, and duration of disease are not limited.

#### 2.1.3. Therapeutic measures

The control group was treated with routine clinical treatment after AMI (including antimyocardial ischemia, statin therapy, anticoagulation, etc), and the experimental group was treated with the auxiliary therapy of Class 1 traditional Chinese medicine injection on the basis of the control group.

#### 2.1.4. Outcome indicators

The primary outcome indexes included: clinical treatment efficiency, the efficacy judgment referred to the “Clinical Disease Diagnosis and Efficacy Judgment Criteria,”^[6]^ “Diagnostic and Therapeutic Guidelines for AMI”^[7]^ and so on; the occurrence of adverse drug reactions; and the secondary endpoints included: left ventricular ejection fraction (LVEF), left ventricular end-diastolic diameter (LVEDD), left ventricular end-systolic internal diameter (LVESD), left ventricular end-diastolic volume (LVEDV), and left ventricular end-systolic volume (LVESV). The differences in pretreatment outcome indices were not statistically significant in any of the groups.

### 2.2. Exclusion criteria

Literature that did not report the above outcome metrics; duplications or the presence of data that were clearly inconsistent; animal studies, reviews, conference reports, registry documents, and non-RCTs; literature that did not meet the target population for treatment or was comorbid with other unrelated diseases; and interventions that were herbal injections combined with other unconventional treatments or were other non-herbal injectable medications.

### 2.3. Search strategy

A comprehensive computer search was conducted across a range of Chinese and English databases, including the China National Knowledge Infrastructure, the Chongqing VIP Database (VIP), the Wanfang Database, the SinoMed Database, PubMed, the Web of Science Database, EMBASE, and the Cochrane Library. The search spanned the period from the database’s inception to April 7, 2024. The search strategy was tailored to the specific characteristics of each database. The database employs a corresponding retrieval strategy. The Chinese search terms include: AMI, myocardial infarction, AMI, acute coronary syndrome, myocardial remodeling, myocardial reconstruction, cardiac reconstruction, myocardial remodeling, myocardial fibrosis, injection liquid, injection solution, extract, Chinese injection liquid, RCT. The English search terms include, but are not limited to, AMI, ventricular remodeling, ventricular remodelings, ventricle remodeling, remodeling, left ventricle, injections, extract, injectable, RCT, clinical observation, and placebo, etc. The method of subject words combined with free words was adopted, and the specific retrieval strategy of each database was shown in the additional materials published in this paper.

### 2.4. Literature screening and data extraction

The final search results were imported into EndNote X9 software, and duplicates were excluded and then the literature was initially screened and rescreened. Then 2 researchers independently screened the literature and extracted the data to determine the final inclusion of the literature, such as encountering differences in the third-party negotiation assessment processing. The content of the data included the following: basic information of the literature (name of the first author, years of experience, etc.), characteristics of the observation subjects (gender, cardiac function classification, etc), therapeutic measures (types of injections, duration of use, etc), and outcome indicators. Excel table was used to standardize the data into a form.

### 2.5. Risk of bias evaluation of the literature

Two separate researchers evaluated the final literature separately using the Cochrane Risk of Bias Assessment Tool and checked the final results.

### 2.6. Statistical analyses

In this study, we performed reticulated Meta-analysis based on the frequency school of thought, and used RevMan5.4 and Stata18.0 MP software for the assessment of risk of bias mapping and reticulated Meta-analysis. Odds ratio was calculated for dichotomous indicators, mean difference (MD) was calculated for continuous indicators, Standardised MD was used if the units or measurements were not standardized, and confidence intervals (credible interval, CI) were displayed using 95% CI. When plotting the reticulated evidence graph, the area of the circles in the graph refers to how much sample size is available for that treatment measure, and the thickness of the lines between the circles refers to the amount of literature included. Inconsistency tests were selected based on the presence or absence of closed loops in the reticulation evidence plot, and if not, analyses were conducted using consistency tests. SUCRA values were calculated and ranked according to the difference in efficacy across different interventions. Finally, a corrected funnel plot was drawn to evaluate the presence of publication bias, too small a sample effect, etc.

## 3. Results

### 3.1. Literature search

**Figure 1.**
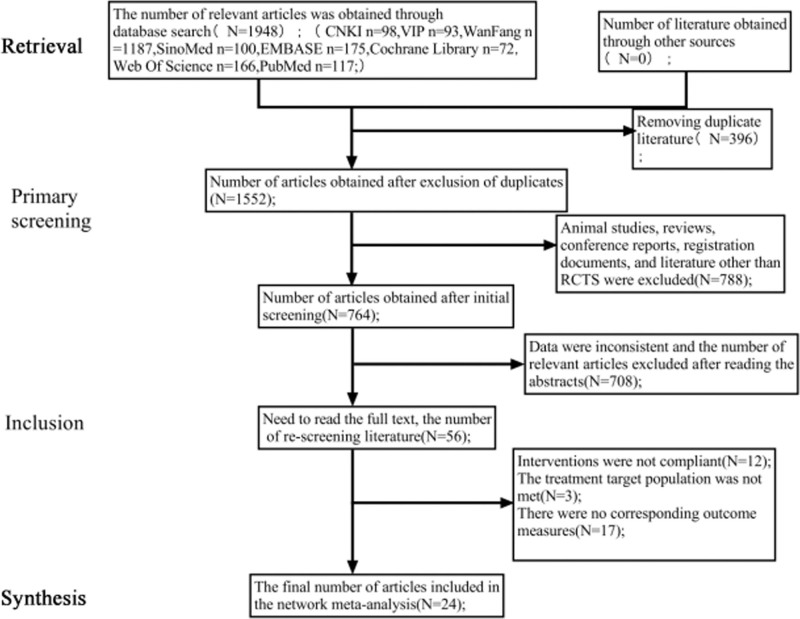
Procedure of literature search and screening.

The retrieved 1948 relevant literatures were managed by EndNote X9 software, and 1552 literatures were remaining by eliminating duplicates, and then 24 literatures, all in Chinese, were finally included after screening according to the inclusion and exclusion criteria. It involved 2343 patients and 12 types of Chinese medicine injections. The comparison of all descriptive information in each group of literature met statistical significance (*P* <.05). The search results and screening process are shown in Figure 1.

### 3.2. Basic characteristics of the included literature

A total of 2343 patients were included in the 24 final included papers,^[8–31]^ with a total of 1175 and 1168 patients in the experimental and control groups, respectively. Twelve types of TCM injections were involved, with a total of 7 outcome indicators: 6 reported clinical treatment efficiency, 14 reported the occurrence of adverse reactions, 24 reported LVEF, 15 reported LVEDV and LVESV, and 14 reported LVEDD and LVESD. The basic characteristics of the included literature are shown in Table 1.

**Table 1:** Basic characteristics of the included literature.

Included literature	Sample size/case	Male/female/case	Mean age/year	Classification of cardiac function (Killip/NYHA)	Interventions	Course of treatment	Outcome measures
	T/C	T/C	T/C	T/C			
Wang Xiaohong, 2015^[8]^	30/30	15/15 14/16	75.00 ± 6.00 74.00 ± 5.00	N:Ⅱ–Ⅳ	SF (1ml·kg^-1^·d^-1^) +CT	15 d	①⑥⑦
Feng Qingli, 2011^[9]^	37/31	20/17 17/14	60.50	/	SF (50 mL·d^−1^) +CT	14 d	①②③⑥⑦
Yan Hua, 2017^[10]^	40/40	22/18 21/19	61.68 ± 7.54 62.03 ± 7.66	K: Ⅰ–Ⅲ	SF (50 mL·d^−1^) +CT	21 d	①②③⑤⑥⑦
Bao Bohang, 2020^[11]^	38/36	32/6 27/9	57.92 ± 12.36 61.75 ± 12.69	K: Ⅰ–Ⅲ	SF (50mL·d^−1^) +CT	10 d	①④⑤⑥⑦
Zhang Sufang, 2009^[12]^	20/20	13/7 11/9	60.90 ± 8.80 60.10 ± 9.06	/	SM (120 mL·d^−1^) +CT	28 d	①②③⑦
Luo Qi, 2020^[13]^	45/45	23/22 26/19	59.87 ± 6.24 59.16 ± 6.37	/	SX (100 mL·d^−1^) +CT	14 d	①②③④⑤⑥⑦
Xu Kai, 2018^[14]^	70/70	34/36 35/35	47.00 ± 11.00 48.00 ± 9.00	/	SX + CT	1 m	①②③④⑦
Yang Jun, 2006^[15]^	40/42	31/9 35/7	59.90 ± 13.10 59.90 ± 13.10	/	CXQ (first 200 mL·d^−1^, then160 mL·d^−1^) +CT	15 d	①②③
Chen Hao, 2017^[16]^	49/49	27/22 29/20	51.20 ± 5.40 49.70 ± 5.70	/	CXQ (120 mg·d^−1^) + CT	3 m	①③⑥
Liu Yang, 2010^[17]^	30/30	21/9 22/8	64.61 ± 8.22 65.23 ± 7.56	K: ≥Ⅱ: 6 ≥Ⅱ: 5	DSDFSY (400 mg·d^−1^) +CT	7 d	①②③④
Peng Fang, 2015^[18]^	45/45	22/23 21/24	56.50 ± 3.30 58.20 ± 2.50	K: Ⅰ–Ⅱ	DH (30 mL·d^−1^) +CT	14 d	①②③④⑥⑦
Qing Niming, 2014^[19]^	56/56	31/25 30/26	52.31 ± 11.24 55.12 ± 10.52	K: Ⅰ–Ⅳ	DH (40 mL·d^−1^) +CT	7 d	①②③④
Li Cui, 2020^[20]^	86/80	45/41 40/40	59.25 ± 6.69 58.75 ± 5.68	K:Ⅱ–Ⅳ	DH (4mL·d^−1^) +CT	14 d	①②③④
Liu Boxin, 2024^[21]^	48/47	29/19 24/23	51.98 ± 7.84 51.44 ± 7.29	K: Ⅰ–Ⅲ	DXGX (20 mL·d^−1^) +CT	28 d	①④⑤⑥
Li Zheng, 2007^[22]^	45/45	30/15 31/14	62.00 ± 15.00 61 ± 14.00	/	GXN (20 mL·d^−1^) +CT	15 d	①②③⑥⑦
Hou Yan, 2012^[23]^	100/106	106/100	57.30 ± 3.50	K: Ⅰ–Ⅲ	HQ (20mL·d^−1^) +CT	28 d	①②③
Zhang Jinguo, 2002^[24]^	54/54	33/21 35/19	60.70 ± 8.20 61.60 ± 9.10	K: Ⅰ–Ⅲ	HQ (20 mL·d^−1^) +CT	28 d	①②③④
Gao Dongsheng, 2002^[25]^	46/46	29/17 28/18	69.20 68.90	/	HQ (20 mL·d^−1^) +CT	28 d	①②③④
Liu Chunxiao, 2016^[26]^	40/40	23/17 21/19	62.50 ± 7.20 63.10 ± 6.90	N:Ⅱ–Ⅳ	SXT (6 mL·d^−1^) +CT	14 d	①⑤⑥⑦
Li Bo, 2018^[27]^	20/20	9/11 8/12	58.26 ± 6.54 59.87 ± 7.54	N:Ⅱ–Ⅳ	SXT (6 mL·d^−1^) +CT	6 mo	①④⑤⑥⑦
Wang Yayun, 2021^[28]^	60/60	42/18 39/21	56.72 ± 6.54 56.67 ± 6.73	/	SXT (8 mL·d^−1^) +CT	28 d	①④⑥⑦
Liu Yanli, 2019^[29]^	76/76	48/28 40/36	61.35 ± 6.72 63.85 ± 6.12	N:Ⅱ–Ⅳ	XML (10 mg·kg^−1^·d^−1^) +CT	10–20 d	①⑥⑦
Shang Wei, 2021^[30]^	40/40	21/19 19/21	60.50 ± 5.20 61.10 ± 5.20	N:Ⅱ–Ⅲ	XXLHN (200 mL·d^−1^) +CT	14 d	①④⑥⑦
Deng Suoqin, 2012^[31]^	60/52	36/24 34/18	56.70 ± 10.20 55.90 ± 11.00	/	SX (200 mL·d^−1^) +CT	28 d	①②③④

CT = conventional therapy, CXQ = Chuanxiong Rhizoma, DH = Dan Hong, DSDFSY = Salvianolate, DXGX = Dan-Xiang Guan Xin, GXN = Guanxin Ning, HQ = Astragalus membranaceus, SF = Shenfu, SM = Shenmai, SXT = Shuxuetong, XML = Xinmailong, XXLHN = Xingxiong sodium chloride.

### 3.3. Evaluation of the quality of the included literature

**Figure 2.**
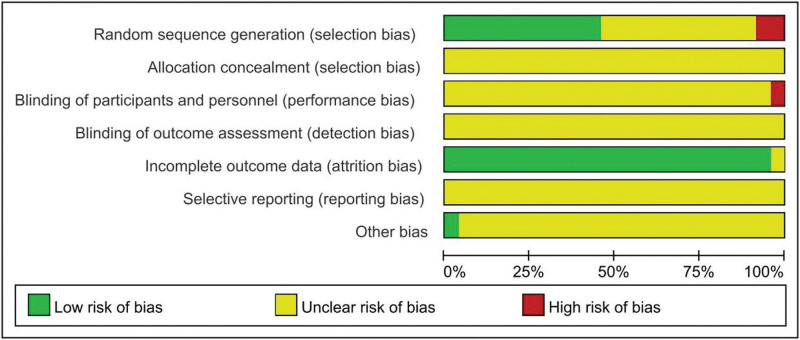
Proportion of literature risk bias risk.

**Figure 3.**
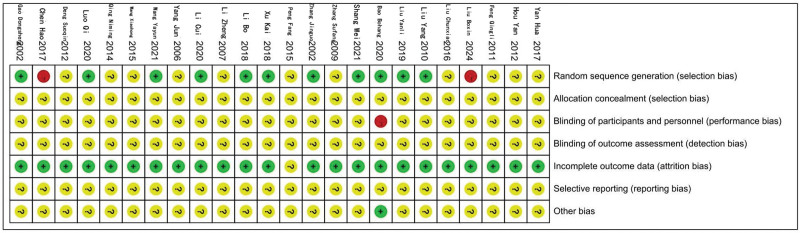
Summary of literature bias risk.

Of the 24 papers included, a total of 11^[11,13,14,17,20–22,27–30]^ used correct randomization methods such as random number tables for the generation of random sequences, 11^[8–10,12,15,18,19,22,23,26,31]^ either did not mention or referred to the fact that they did not describe a specific randomization method, and 2^[16,21]^ had a high level of risk. None of the literature mentioned allocation scheme concealment. In terms of blinding, 1 paper^[11]^ was at high risk and the rest were at uncertain risk of bias. In terms of completeness of outcome indicators, 1^[18]^ literature was at uncertain risk of bias, and the rest were at low risk. All were at uncertain risk of bias in terms of selective reporting of studies. The presence of other sources of bias was shown to be low risk of bias in 1^[11]^ literature and uncertain in the rest. The risk of bias evaluation is shown in Figures 2 and 3.

### 3.4. Reticulated evidence maps

**Figure 4.**
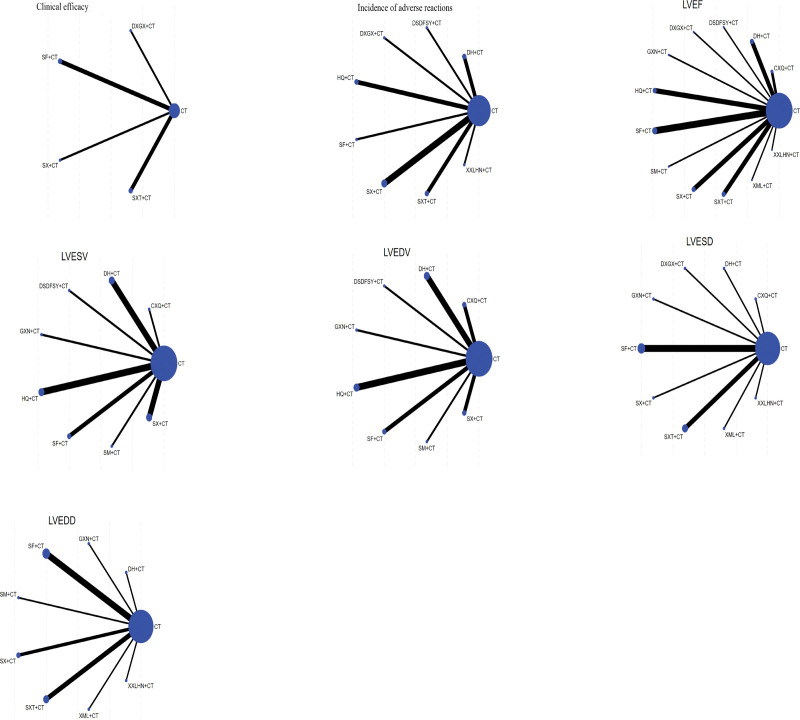
Network evidence plot for each outcome measure. CT = conventional therapy; each injection, CXQ = Chuanxiong Rhizoma, DSDFSY = Salvianolate, DH = Dan Hong, DXGX = Dan-Xiang Guan Xin, GXN = Guanxin Ning, HQ = Astragalus membranaceus, LVEF = left ventricular ejection fraction, LVESV = left ventricular end-systolic volume, LVEDV = left ventricular end-diastolic volume, LVESD = left ventricular end-systolic internal diameter, LVEDD = left ventricular end-diastolic internal diameter, SF = Shenfu, SM = Shenmai, SX = Shenxiong, SXT = Shuxuetong, XML = Xinmailong, XXLHN = Xingxiong sodium chloride.

The final web of evidence for each outcome metric of ventricular remodeling after AMI assisted by 12 herbal injections is shown in Figure 4.

### 3.5. Clinical efficacy

**Figure 5.**
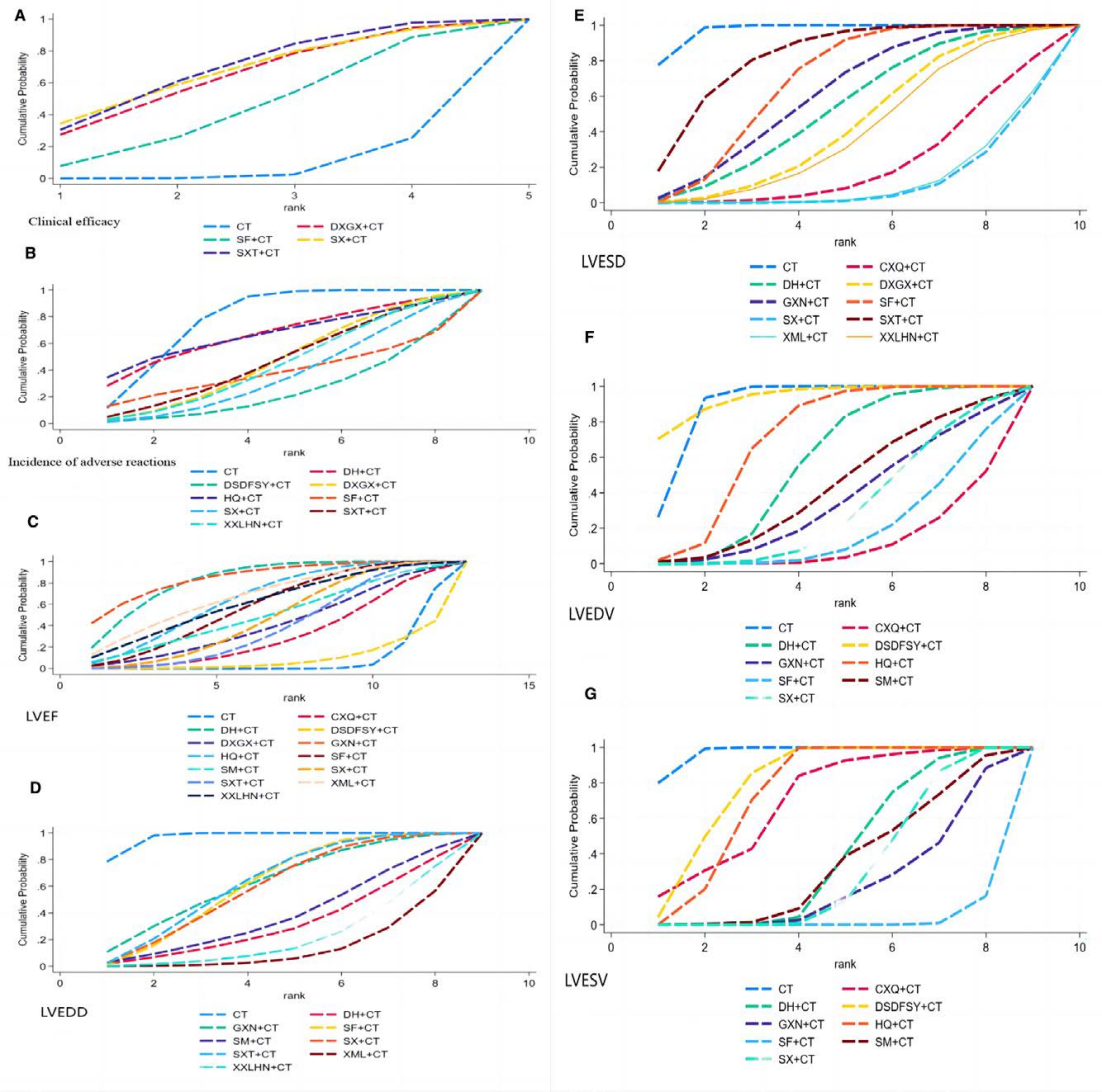
SUCRA probability ranking diagram of each outcome indicator. CT = conventional therapy; each injection, CXQ: Chuanxiong Rhizoma, DH = Dan Hong, DSDFSY = Salvianolate, DXGX = Dan-Xiang Guan Xin, GXN = Guanxin Ning, HQ = Astragalus membranaceus, LVEF = left ventricular ejection fraction;LVESV = left ventricular end-systolic volume, LVEDV = left ventricular end-diastolic volume, LVESD = left ventricular end-systolic internal diameter, LVEDD = left ventricular end-diastolic internal diameter, SF = Shenfu, SM = Shenmai, SX = Shenxiong, SXT = Shuxuetong, XML = Xinmailong, XXLHN = Xingxiong sodium chloride.

The results showed a total of 6^[10,11,13,21,26,27]^ RCTs reporting clinical treatment efficacy involving 4 herbal injections and a total of 5 interventions. The analyses produced a total of 10 comparisons, none of which were statistically significant (*P* >.05). And SUCRA sorting revealed that Shuxuetong injection assisted with conventional therapy (CT) may be the intervention with the best clinical efficacy, of which: Shuxuetong injection + conventional therapy (SUCRA = 68.5%) > Shenxiong injection + conventional therapy (SUCRA = 66.7%) > Dan-Xiang Guan Xin injection + conventional therapy (SUCRA = 63.7%) > Shenfu injection + conventional therapy (SUCRA = 44.1%) > Conventional therapy (SUCRA = 7.0%); (for details, see Figure 5A, Table 2).

**Table 2:** Results of network meta-analysis of clinical efficacy (OR, [95% CI]).

Interventions	CT	DXGX + CT	SF + CT	SX + CT	SXT + CT
CT	0	/	/	/	/
DXGX + CT	0.27 (0.05, 1.44)	0	/	/	/
SF + CT	0.46 (0.12, 1.71)	1.66 (0.20, 13.83)	0	/	/
SX + CT	0.25 (0.04, 1.56)	0.91 (0.08, 10.79)	0.55 (0.06, 5.25)	0	/
SXT + CT	0.25 (0.06, 1.03)	0.92 (0.10, 8.10)	0.55 (0.08, 3.82)	1.00 (0.10, 10.19)	0

CI = confidence interval, CT = conventional therapy, CXQ = Chuanxiong Rhizoma, DH = Dan Hong, DXGX = Dan-Xiang Guan Xin, OR = odds ratio, SF = Shenfu, SX = Shenxiong, SXT = Shuxuetong.

### 3.6. Incidence of adverse reactions

The results showed a total of 14^[11,13,14,17–21,24,25,27,28,30,31]^ RCTs reported the occurrence of adverse reactions, which were related to the presence of sudden cardiogenic death, heart failure, angina pectoris, gastrointestinal systemic adverse reactions, and skin rash (see Table 3 for details). There were 8 herbal injections included, for a total of 9 interventions. The analyses produced a total of 36 comparisons, and the results of the differences between the comparisons were not statistically significant (*P* >.05). And SUCRA sorting saw that Salvianolate injection adjunctive conventional therapy may be the intervention with the least occurrence of adverse reactions and the best safety profile, including: Conventional therapy (SUCRA = 78.4%) > Danhong injection + conventional therapy (SUCRA = 67.0%) > Astragalus membranaceus injection + conventional therapy (SUCRA = 66.8%) > Shuxuetong injection + conventional therapy (SUCRA = 47.1%)>Dan-xiang Guan Xin injection + conventional therapy (SUCRA = 46.9%)>Xingxiong sodium chloride injection + conventional therapy (SUCRA = 44.1%)>Shenfu injection + conventional Therapy (SUCRA = 38.5%)>Shenxiong injection + conventional therapy(SUCRA = 36.6%)>Salvianolate injection + conventional therapy (SUCRA = 24.6%); (see Fig. 5B and Table 4 for details).

**Table 3:** Occurrence of adverse reactions.

Included literature	Interventions	Occurrence of adverse reactions
		T/C
Bao Bohang, 2020^[11]^	SF + CT	One case of sudden cardiac death
Luo Qi, 2020^[13]^	SX + CT	/
Xu Kai, 2018^[14]^	SX + CT	A case of hypertension/a case of hypertension
Liu Yang, 2010^[17]^	DSDFSY + CT	Two patients had severe heart failure/7 patients had severe heart failure
Peng Fang, 2015^[18]^	DH + CT	There were 3 cases of heart failure, 3 of angina pectoris, 6 of cardiac events, 2 of bleeding, and 2 of complications/there was 1 cardiac death, 1 combined endpoint, 5 heart failure, 4 angina, 11 cardiac events, 1 bleeding, and 1 complication
Qing Niming, 2014^[19]^	DH + CT	One case had mild gastrointestinal adverse reaction/1 case had mild gastrointestinal adverse reaction
Li Cui, 2020^[20]^	DH + CT	There was 1 case of gingival bleeding, 3 cases of rash, and 1 case of diarrhea/ There was 1 case of gingival bleeding, 4 cases of gastrointestinal bleeding, 1 case of rash, 2 cases of dyspnea, and 1 case of diarrhea
Liu Boxin, 2024^[21]^	DH + CT	/
Zhang Jinguo, 2002^[24]^	HQ + CT	/
Gao Dongsheng, 2002^[25]^	HQ + CT	/
Li Bo, 2018^[27]^	SXT + CT	There was 1 case of allergy and 1 case of nausea and vomiting/ There was 1 case of myocardial infarction, 1 case of abnormal liver and kidney function, and 1 case of nausea and vomiting
Wang Yayun, 2021^[28]^	SXT + CT	A case of rash/there was 2 cases of pruritus and 1 case of palpitations
Shang Wei, 2021^[30]^	XXLHN + CT	There was 1 myocardial infarction, 1 cardiogenic shock, and 2 heart failure/there were 4 cases of myocardial infarction, 3 cases of noncardiogenic shock, and 1 case of heart failure
Deng Suoqin, 2012^[31]^	SX + CT	One case of heart failure and one of malignant arrhythmia/there were 2 cases of angina, 3 cases of malignant arrhythmia, and 3 cases of heart failure

CT = conventional therapy, CXQ = Chuanxiong Rhizoma, DH = Dan Hong, DSDFSY = Salvianolate, GXN = Guanxin Ning, HQ = Astragalus membranaceus, SF = Shenfu, SM = Shenmai, SX = Shenxiong, SXT = Shuxuetong, XXLHN = Xingxiong sodium chloride.

**Table 4:** Results of network meta-analysis of adverse reactions (OR, [95% CI]).

Interventions	CT	DH + CT	DSDFSY + CT	DXGX + CT	HQ + CT	SF + CT	SX + CT	SXT + CT	XXLHN + CT
CT	0	/	/	/	/	/	/	/	/
DH + CT	1.05 (0.11, 10.23)	0	/	/	/	/	/	/	/
DSDFSY + CT	4.26 (0.81, 22.53)	4.06 (0.24, 68.13)	0	/	/	/	/	/	/
DXGX + CT	2.04 (0.63, 6.61)	1.94 (0.15, 25.16)	0.48 (0.06, 3.67)	0	/	/	/	/	/
HQ + CT	1.00 (0.06, 16.21)	0.95 (0.03, 34.77)	0.23 (0.01, 6.03)	0.49 (0.02, 10.10)	0	/	/	/	/
SF + CT	3.25 (0.13, 82.49)	3.10 (0.06, 161.58)	0.76 (0.02, 28.99)	1.60 (0.05, 49.84)	3.25 (0.05, 232.11)	0	/	/	/
SX + CT	2.72 (0.73, 10.18))	2.59 (0.19, 36.00)	0.64 (0.08, 5.35)	1.34 (0.23, 7.83)	2.72 (0.12, 59.35)	0.84 (0.03, 27.47)	0	/	/
SXT + CT	2.09 (0.48, 9.06)	1.99 (0.13, 29.85)	0.49 (0.05, 4.51)	1.03 (0.16, 6.73)	2.09 (0.09, 48.67)	0.64 (0.02, 22.36)	0.77 (0.11, 5.52)	0	/
XXLHN + CT	2.25 (0.62, 8.18)	2.14 (0.16, 29.35)	0.53 (0.06, 4.34)	1.10 (0.19, 6.34)	2.25 (0.10, 48.48)	0.69 (0.02, 22.48)	0.83 (0.13, 5.24)	1.08 (0.15, 7.60)	0

CI = confidence interval, CT = conventional therapy, CXQ = Chuanxiong Rhizoma, DH = Dan Hong, DSDFSY = Salvianolate, DXGX = Dan-Xiang Guan Xin, GXN = Guanxin Ning, HQ = Astragalus membranaceus, LVEF = left ventricular ejection fraction, OR = odds ratio, SF = Shenfu, SM = Shenmai, SX = Shenxiong, SXT = Shuxuetong, XML = Xinmailong, XXLHN = Xingxiong sodium chloride.

### 3.7. LVEF

The results showed a total of 24^[8–31]^ RCTs reporting on LVEF, involving 12 of all TCM injections, for a total of 13 interventions. The analysis produced 78 comparisons and 10 comparisons with statistically significant differences (*P* <.05); Danhong injection (MD = −8.97, 95% CI [−12.88, −5.06]), GuanxinNing injection (MD = −10.00,95% CI [−16.57, −3.43]), Astragalus membranaceus injection (MD = −6.79. 95%CI [−11.05, −2.53]), Shenfu Injection (MD = −6.02, 95% CI [−10.45, −1.59]), Shenxiong Injection (MD = −4.93, 95% CI [−8.83, −1.04]), Shuxuetong Injection (MD = −4.01, 95% CI [−7.92, −0.11]) (MD = −7.27, 95% CI [−13.76, −0.78]), the above injections were significantly better than conventional therapy alone in enhancing LVEF levels with ventricular remodeling after acute infarction. In the comparison of LVEF enhancement among injections, Danhong injection was significantly superior to Danshen polyphenate injection (MD = 9.97, 95% CI [2.43,17.52]), Dan-xiang Guan injection was significantly superior to Salvianolate injection (MD = -11.00, 95% CI [-20.21,-1.79]), Astragalus membranaceus injection was significantly superior to Salvianolate injection (MD = -7.79,95%CI [-15.53,-0.05]); and SUCRA sorting revealed that Guanxin Ning injection adjunctive to conventional therapy might be the best intervention to enhance the LVEF level, among which: Guanxin Ninginjection + conventional therapy (SUCRA = 85.2%) > Danhong injection + conventional therapy (SUCRA = 82.9%) > Xinmailong injection + conventional therapy (SUCRA = 67.7%) > Astragalus membranaceus injection + conventional therapy (SUCRA = 65.2%) > Xingxiong sodium chloride injection + conventional therapy (SUCRA = 61.9%) > Shenfu injection + conventional therapy (SUCRA = 58.4%) > Shenmai injections + conventional therapy (SUCRA = 50.2%) > Shenxiong injection + conventional therapy (SUCRA = 47.2%) therapy (SUCRA = 47.7%)>Dan-xiang Guan Xin injection + conventional therapy (SUCRA = 41.7%)> Shuxuetong injection + conventional therapy (SUCRA = 39.7%)> Chuanxiong Rhizoma injection + conventional therapy (SUCRA = 31.3%)> Salvianolate injection + conventional therapy (SUCRA = 9.6%)> Conventional therapy (SUCRA = 8.6%); (see Fig. 5C for details, Table 5).

**Table 5:** Results of network Meta-analysis of LVEF (MD, [95% CI]).

Interventions	CT	CXQ + CT	DH + CT	DSDFSY + CT	DXGX + CT	GXN + CT	HQ + CT	SF + CT	SM + CT	SX + CT	SXT + CT	XML + CT	XXLHN + CT
CT	0	/	/	/	/	/	/	/	/	/	/	/	/
CXQ + CT	-2.88 (−8.26, 2.50)	0	/	/	/	/	/	/	/	/	/	/	/
DH + CT	−8.97 (−12.88, −5.06)	−6.09 (−12.76, 0.57)	0	/	/	/	/	/	/	/	/	/	/
DSDFSY + CT	1.00 (−5.46, 7.46)	3.88 (−4.53, 12.29)	9.97 (2.43, 17.52)	0	/	/	/	/	/	/	/	/	/
DXGX + CT	−4.06 (−10.49, 2.37)	−1.18 (−9.56, 7.20)	4.91 (−2.61, 12.44)	−5.06 (−14.17, 4.05)	0	/	/	/	/	/	/	/	/
GXN + CT	−10.00 (−16.57, −3.43)	−7.12 (−15.61, 1.37)	−1.03 (−8.67, 6.61)	−11.00 (−20.21, −1.79)	−5.94 (−15.13, 3.25)	0	/	/	/	/	/	/	/
HQ + CT	−6.79 (−11.05, −2.53)	−3.91 (−10.79, 2.97)	2.18 (−3.59, 7.96)	−7.79 (−15.53, −0.05)	−2.73 (−10.44, .98)	3.21 (−4.62, 11.04)	0	/	/	/	/	/	/
SF + CT	−6.02 (−10.45, −1.59)	−3.14 (−10.12, 3.85)	2.95 (−2.95, 8.85)	−7.02 (−14.85, 0.81)	−1.96 (−9.77, 5.85)	3.98 (−3.94, 11.90)	0.77 (−5.37, 6.91)	0	/	/	/	/	/
SM + CT	−5.08 (−12.45, 2.29)	−2.20 (−11.33, 6.93)	3.89 (−4.45, 12.23)	−6.08 (−15.88, 3.72)	−1.02 (−10.80, 8.76)	4.92 (−4.95, 14.79)	1.71 (−6.80, 10.22)	0.94 (−7.66, 9.54)	0	/	/	/	/
SX + CT	−4.93 (−8.83, −1.04)	−2.05 (−8.70, 4.59)	4.04 (−1.48, 9.56)	−5.93 (−13.48, 1.61)	−0.87 (−8.39, 6.64)	5.07 (−2.57, 12.70)	1.85 (−3.92, 7.63)	1.08 (−4.81, 6.98)	0.15 (−8.19, 8.48)	0	/	/	/
SXT + CT	−4.01 (−7.92, −0.11)	−1.13 (−7.77, 5.51)	4.96 (−0.57, 10.49)	−5.01 (−12.56, 2.53)	0.05 (−7.47, 7.57)	5.99 (−1.65, 13.63)	2.78 (−3.01, 8.57)	2.01 (−3.90, 7.92)	1.07 (−7.27, 9.41)	0.92 (−4.59, 6.44)	0	/	/
XML + CT	−7.27 (−13.76, −0.78)	−4.39 (−12.82, 4.04)	1.70 (−5.87, 9.28)	−8.27 (−17.42, .88)	−3.21 (−12.34, 5.92)	2.73 (−6.50, 11.96)	−0.48 (−8.24, 7.28)	−1.25 (−9.11, 6.61)	−2.19 (−12.01, 7.63)	−2.34 (−9.90, 5.23)	−3.26 (−10.83, 4.32)	0	/
XXLHN + CT	−6.60 (−13.46, 0.26)	−3.72 (−12.44, 5.00)	2.37 (−5.53, 10.27)	−7.60 (−17.02, .82)	−2.54 (−11.94, 6.86)	3.40 (−6.10, 12.90)	0.19 (−7.89, 8.27)	−0.58 (−8.75, 7.59)	−1.52 (−11.59, 8.55)	−1.67 (−9.56, 6.23)	−2.59 (−10.49, 5.31)	0.67 (−8.78, 10.12)	0

CI = confidence interval, CT = conventional therapy, CXQ = Chuanxiong Rhizoma, DH = Dan Hong, DSDFSY = Salvianolate, DXGX = Dan-Xiang Guan Xin, GXN = Guanxin Ning, HQ = Astragalus membranaceus, LVEF = left ventricular ejection fraction, MD = mean difference, SF = Shenfu, SM = Shenmai, SX = Shenxiong, SXT = Shuxuetong, XML = Xinmailong, XXLHN = Xingxiong sodium chloride.

### 3.8. LVEDD

The results showed a total of 14^[8–14,18,22,26–30]^ RCTs^[8–14,18,22,26–30]^ reported on left ventricular end-diastolic internal diameter (LVEDD), which involved 8 herbal injections for a total of 9 interventions. The analysis yielded 36 comparisons, and the difference between 4 comparisons was statistically significant (*P* <.05); among them, Danhong injection (MD = 6.00, 95% CI [0.06,11.94]), Shenfu injection (MD = 3.08, 95% CI [0.52,5.63]), and Xinmailong injection (MD = 8.08, 95% CI [3.43,12.73]), Xingxiong sodium chloride Injection (MD = 7.00, 95% CI [2.16,11.84]), the above injections were significantly superior to conventional therapy alone in reducing LVEDD levels by adjunctive ventricular remodeling in the treatment of acute postinfarction. And SUCRA ranking shows that: Xingxiong sodium chloride injection assisted conventional therapy may be the best intervention among them (This is an ascending ranking, and the opposite is true for decreasing the index. LVESD, LVEDV and LVESV were the same. The clinical indexes of LVEDD/LVESD/LVEDV/LVESV were better if they were reduced): Conventional therapy (SUCRA = 97.1%) > Shuxuetong injection + conventional therapy (SUCRA = 63.2%) > Guanxin Ning injection + conventional therapy (SUCRA = 63.1%)>Shenfu injection + conventional therapy (SUCRA = 61.6%)>Shenxiong injection + conventional therapy (SUCRA = 59.4%)>Shenmai injection + conventional therapy (SUCRA = 38.1%) > Danhong injection + conventional therapy (SUCRA = 32.1%)>Xingxiong sodium chloride injection + conventional therapy (SUCRA = 21.9%)>Xinmailong injection + conventional therapy (SUCRA = 13.6%); (see Fig. 5D and Table 6 for details).

**Table 6:** Results of network meta-analysis of LVEDD (MD, [95% CI]).

Interventions	CT	DH + CT	GXN + CT	SF + CT	SM + CT	SX + CT	SXT + CT	XML + CT	XXLHN + CT
CT	0	/	/	/	/	/	/	/	/
DH + CT	6.00 (0.06, 11.94)	0	/	/	/	/	/	/	/
GXN + CT	2.89 (−1.75, 7.53)	−3.11 (−10.65, 4.43)	0	/	/	/	/	/	/
SF + CT	3.08 (0.52, 5.63)	−2.92 (−9.39, 3.55)	0.19 (−5.11, 5.49)	0	/	/	/	/	/
SM + CT	5.35 (−0.22, 10.92)	−0.65 (−8.80, 7.50)	2.46 (−4.80, 9.72)	2.27 (−3.86,8.40)	0	/	/	/	/
SX + CT	3.30 (−0.12, 6.72)	−2.70 (−9.56, 4.15)	0.41 (−5.36, 6.18)	0.22 (−4.06, 4.50)	−2.05 (−8.59, 4.49)	0	/	/	/
SXT + CT	2.92 (−0.11, 5.95)	−3.08 (−9.75, 3.59)	0.03 (−5.51, 5.58)	−0.15 (−4.12, 3.81)	−2.43 (−8.77, 3.92)	−0.38 (−4.94, 4.19)	0	/	/
XML + CT	8.08 (3.43, 12.73)	2.08 (−5.46, 9.62)	5.19 (−1.38, 11.76)	5.00 (−0.30, 10.31)	2.73 (−4.53, 9.99)	4.78 (−0.99, 10.55)	5.16 (−0.39, 10.71)	0	/
XXLHN + CT	7.00 (2.16, 11.84)	1.00 (−6.66, 8.66)	4.11 (−2.60, 10.82)	3.92 (−1.55, 9.39)	1.65 (−5.73, 9.03)	3.70 (−2.22, 9.63)	4.08 (−1.63, 9.79)	−1.08 (−7.79, 5.63)	0

CI = confidence interval, CT = conventional therapy, CXQ = Chuanxiong Rhizoma, DH = Dan Hong, GXN = Guanxin Ning, HQ = Astragalus membranaceus, LVEDD = left ventricular end-diastolic internal diameter, MD = mean difference, SF = Shenfu, SM = Shenmai, SXT = Shuxuetong, XML = Xinmailong, XXLHN = Xingxiong sodium chloride.

### 3.9. LVESD

The results showed a total of 14 [8–11, 13, 16, 18, 21–22, 26–30] RCTs reported on left ventricular end-systolic internal diameter (LVESD), which involved 9 herbal injections for a total of 10 interventions. The analysis yielded 45 comparisons, and the differences were statistically significant in 13 comparisons (*P* <.05); among them, Chuanxiong Rhizoma injection (MD = 7.06, 95% CI [3.09,11.03]), Danhong injection (MD = 4.00,95% CI [0.21,7.79]), Dan-xiang Guan Xin injection (MD = 4.82,95% CI [1.38,8.26]), Shenfu Injection (SMD = 2.79,95%CI [0.77,4.81]), Shenxiong Injection (MD = 8.33,95%CI [5.01,11.65]), Xinmailong Injection (MD = 8.21,95%CI [4.82,11.60]), Xingxiong sodium chloride Injection (MD = 5.20, 95% CI [1.62,8.78]), the above injections were significantly superior to conventional therapy alone in reducing LVESD levels with adjunctive ventricular remodeling after acute infarction. In the comparison of LVESD reduction among injections, Chuanxiong Rhizoma was significantly superior to Shuxuetong injection (MD = -5.64, 95% CI [-10.66,-0.62]), Shenxiong was significantly superior to Guanxin Ning injection (MD = 4.95, 95% CI [0.14,9.76]), and Shenxiong was significantly superior to Shenfu injection (MD = 5.54, 95% CI [1.65,9.43]), Xinmailong was significantly superior to Shenfu injection (MD = 5.42, 95% CI [1.47,9.36]), Shenxiong was significantly superior to Shuxuetong injection (MD = -6.91, 95% CI [-11.43,-2.38]), and Xinmailong was significantly superior to Shuxuetong injection (MD = 6.79, 95% CI [2.21, 11.36]); and SUCRA ranking showed that Shenxiong Injection supplemented with conventional therapy might be the best intervention among them, in which: Conventional therapy (SUCRA = 97.4%) > Shuxuetong injection + conventional therapy (SUCRA = 82.7%) > Shenfu Injection + conventional therapy (SUCRA = 69.4%) > Guanxin Ning injection + conventional therapy (SUCRA (SUCRA = 62.2%)>Danhong Injection + conventional therapy (SUCRA = 54.6%)>Dan-xiang Guan Xin Injection + conventional therapy (SUCRA = 45.3%) > Xingxiong sodium chloride Injection + conventional therapy (SUCRA = 41.4%) > Chuanxiong Rhizoma Injection + conventional therapy (SUCRA = 22.8%)>Xinmailong Injection + conventional therapy (SUCRA = 12.6%) 12.6%) > Shenxiong Injection + conventional therapy (SUCRA = 11.6%); (see Fig. 5E and Table 7 for details).

**Table 7:** Results of network meta-analysis of LVESD (MD, [95% CI]).

Interventions	CT	CXQ + CT	DH + CT	DXGX + CT	GXN + CT	SF + CT	SX + CT	SXT + CT	XML + CT	XXLHN + CT
CT	0	/	/	/	/	/	/	/	/	/
CXQ + CT	7.06 (3.09, 11.03)	0	/	/	/	/	/	/	/	/
DH + CT	4.00 (0.21, 7.79)	−3.06 (−8.55, 2.43)	0	/	/	/	/	/	/	/
DXGX + CT	4.82 (1.38, 8.26)	−2.24 (−7.49, 3.01)	0.82 (−4.30, 5.94)	0	/	/	/	/	/	/
GXN + CT	3.38 (−0.10, 6.86)	−3.68 (−8.96, 1.60)	−0.62 (−5.76, 4.52)	−1.44 (−6.33, 3.45)	0	/	/	/	/	/
SF + CT	2.79 (0.77, 4.81)	−4.27 (−8.72, 0.18)	−1.21 (−5.51, 3.09)	−2.03 (−6.02, 1.96)	−0.59 (−4.61, 3.43)	0	/	/	/	/
SX + CT	8.33 (5.01, 11.65)	1.27 (−3.90, 6.44)	4.33 (−0.71, 9.37)	3.51 (−1.27, 8.29)	4.95 (0.14, 9.76)	5.54 (1.65, 9.43)	0	/	/	/
SXT + CT	1.42 (−1.65, 4.50)	−5.64 (−10.66, −0.62)	−2.58 (−7.46, 2.31)	−3.40 (−8.01, 1.22)	−1.96 (−6.60, 2.69)	−1.37 (−5.05, 2.32)	−6.91 (−11.43, −2.38)	0	/	/
XML + CT	8.21 (4.82, 11.60)	1.15 (−4.07, 6.37)	4.21 (−0.88, 9.30)	3.39 (−1.44, 8.22)	4.83 (−0.03, 9.69)	5.42 (1.47, 9.36)	−0.12 (−4.86, 4.62)	6.79 (2.21, 11.36)	0	/
XXLHN + CT	5.20 (1.62, 8.78)	−1.86 (−7.20, 3.48)	1.20 (−4.01, 6.41)	0.38 (−4.58, 5.34)	1.82 (−3.17, 6.81)	2.41 (−1.70, 6.52)	−3.13 (−8.01, 1.75)	3.78 (−8.01, 1.75)	−3.01 (−7.94, 1.92)	0

CI = confidence interval, CT = conventional therapy, CXQ = Chuanxiong Rhizoma, DH = Dan Hong, DXGX = Dan-Xiang Guan Xin, GXN = Guanxin Ning, HQ = Astragalus membranaceus, LVESD = left ventricular end-systolic internal diameter, MD = mean difference, SF = Shenfu, SM = Shenmai, SXT = Shuxuetong, XML = Xinmailong, XXLHN = Xingxiong sodium chloride.

### 3.10. LVEDV

The results showed a total of 15^[9,10,12,14–20,22–25,31]^ RCTs reported on LVEDV, which involved 8 herbal injections, totaling 9 interventions. The analysis yielded 36 comparisons, and the differences were statistically significant in 13 comparisons (*P* <.05); among them, Chuanxiong Rhizoma injection (MD = 21.78, 95% CI [11.97,31.59]), Danhong injection (MD = 9.36, 95% CI [2.45,16.28]), and Guanxin Ning injection (MD = 15.16, 95% CI [1.80,28.52]), Shenfu Injection (MD = 19.14,95%CI [10.00,28.28]), Shenmai Injection (MD = 13.05,95%CI [0.20,25.90]), Shenxiong Injection (MD = 16.07,95%CI [7.08,25.06]), and the above injections supplemented the routine treatment of acute postinfarction ventricular remodeling was significantly superior to conventional therapy alone in reducing LVEDV levels. In the comparison of LVEDV reduction among injections, Chuanxiong Rhizoma was significantly superior to Danhong injection (MD = -12.42, 95% CI [-14.33,-0.51]), Chuanxiong Rhizoma was significantly superior to Salvianolate injection (MD = -25.19, 95% CI [-40.36,-10.02]), and Chuanxiong Rhizoma was significantly superior to Astragalus membranaceus injection (MD=- 16.25, 95% CI [-10.02]). 16.25, 95% CI [-28.12,-4.38]), Guanxin Ning was significantly superior to Salvianolate injection (MD = 18.57, 95% CI [0.89,36.25]), Shenfu were significantly superior to Salvianolate injection (MD = 22.55, 95% CI[7.80,37.30]), and Shenxiong were significantly superior to Salvianolate injection (MD = 19.48, 95% CI [4.82,34.14]), and Shenfu were significantly better than Astragalus membranaceus injection (MD = 13.61, 95% CI [2.27,24.96]); and SUCRA ranking showed that: Chuanxiong Rhizoma injection adjunctive to conventional therapy might be the best intervention among them, among which: Salvianolate injection + conventional therapy (SUCRA = 93.8%) > Conventional therapy (SUCRA = 89.9%) > Astragalus membranaceus injection + conventional therapy (SUCRA = 70.6%) > Danhong injection + conventional therapy (SUCRA = 56.5%) > shenmai injection + conventional therapy (SUCRA = 42.5%) > Guanxin Ning injection + conventional therapy (SUCRA = 35.0%) > Shenxiong Injection + conventional Therapy (SUCRA = 30.9%) > Shenfu Injection + conventional Therapy (SUCRA = 19.1%) > Chuanxiong Rhizoma Injection + conventional Therapy (SUCRA = 11.7%); (see Fig. 5F for details, Table 8).

**Table 8:** Results of network meta-analysis of LVEDV (MD, [95% CI]).

Interventions	CT	CXQ + CT	DH + CT	DSDFSY + CT	GXN + CT	HQ + CT	SF + CT	SM + CT	SX + CT
CT	0	/	/	/	/	/	/	/	/
CXQ + CT	21.78 (11.97, 31.59)	0	/	/	/	/	/	/	/
DH + CT	9.36 (2.45, 16.28)	−12.42 (−24.33, −0.51)	0	/	/	/	/	/	/
DSDFSY + CT	−3.41 (−14.99, 8.17)	−25.19 (−40.36, −10.02)	−12.77 (−26.26, 0.71)	0	/	/	/	/	/
GXN + CT	15.16 (1.80, 28.52)	−6.62 (−23.19, 9.95)	5.80 (−9.24, 20.83)	18.57 (0.89, 36.25)	0	/	/	/	/
HQ + CT	5.53 (−1.19, 12.25)	−16.25 (−28.12, −4.38)	−3.83 (−13.47, 5.80)	8.94 (−4.45, 22.33)	−9.63 (−24.58, 5.33)	0	/	/	/
SF + CT	19.14 (10.00, 28.28)	−2.64 (−16.03, 10.76)	9.78 (−1.68, 21.24)	22.55 (7.80, 37.30)	3.98 (−12.20, 20.17)	13.61 (2.27, 24.96)	0	/	/
SM + CT	13.05 (0.20, 25.90)	−8.73 (−24.89, 7.43)	3.69 (−10.90, 18.27)	16.46 (−0.83, 33.75)	−2.11 (−20.64, 16.42)	7.52 (−6.98, 22.02)	−6.09 (−21.86, 9.67)	0	/
SX + CT	16.07 (7.08, 25.06)	−5.71 (−18.84, 7.42)	6.71 (−4.60, 18.01)	19.48 (4.82, 34.14)	0.91 (−15.19, 17.01)	10.54 (−0.68, 21.76)	−3.07 (−15.89, 9.74)	3.02 (−12.66, 18.70)	0

CI = confidence interval, CT = conventional therapy, CXQ = Chuanxiong Rhizoma, DH = Dan Hong, DSDFSY = Salvianolate, GXN = Guanxin Ning, HQ = Astragalus membranaceus, LVEDV = left ventricular end-diastolic volume, MD = mean difference, SF = Shenfu, SM = Shenmai, SX = Shenxiong.

### 3.11. LVESV

The results showed a total of 15^[9,10,12–15,17–20,22–25,31]^ RCTs reported on LVESV, which involved 8 herbal injections, totaling 9 interventions. The analysis yielded 36 comparisons, and the differences were statistically significant in 19 comparisons (*P* <.05); among them, Danhong injection (MD = 9.00, 95% CI [6.71,11.29]), Guanxin Ning injection (MD = 11.23, 95% CI [5.71,16.75]), Astragalus membranaceus injection (MD = 2.68, 95% CI [1.38,3.99]), Shenfu Injection (MD = 14.90,95%CI [12.62,17.18]), Shenmai Injection (MD = 9.50,95%CI [3.48,15.52]), and Shenxiong Injection (MD = 9.79,95%CI [8.21,11.37]), the above injections assisted the conventional treatment of acute postinfarction Ventricular remodeling reduced LVESV levels significantly better than conventional therapy alone. In the comparison of LVESV reduction among the injections, Shenfu was significantly superior to Chuanxiong Rhizoma injection (MD = 11.30, 95% CI [3.77,18.83]), Danhong was significantly superior to Salvianolate injection (MD = -7.01, 95% CI [-10.30,-3.71]), and Danhong was significantly superior to Astragalus membranaceus injection (MD = -6.31, 95% CI [-8.95]). [-8.95,-3.68]), Shenfu was significantly superior to Danhong injection (MD = 5.90, 95% CI [2.67,9.14]), Guanxin Ning was significantly superior to Salvianolate injection (MD = 9.24, 95% CI [3.23,15.25]), Shenfu was significantly superior to Salvianolate injection (MD = 12.91, 95% CI [9.62,16.20]), Shenmai significantly superior to Salvianolate injection (MD = 7.51, 95% CI [1.04,13.98]), Shenxiong significantly superior to Salvianolate injection (MD = 7.80, 95% CI [4.95,10.65]), and Guanxin Ning significantly superior to Astragalus membranaceus injection (MD = -8.55, 95% CI [- 14.22,-2.87]), Shenfu significantly superior to Astragalus membranaceus injection (MD = 12.22, 95% CI [9.59,14.85]), Shenmai significantly superior to Astragalus membranaceus injection (MD = 6.82, 95% CI [0.65,12.98]), Shenxiong significantly superior to Astragalus membranaceus injection (MD = 7.11, 95% CI [5.06,9.16]) Shenfu were significantly superior to Shenxiong Injection (MD = -5.11, 95% CI [-7.88,-2.33]); and SUCRA ranking showed that Shenfu Injection supplemented with conventional therapy might be the best intervention among them, among which: Conventional therapy (SUCRA = 97.4%) > Salvianolate injection + conventional therapy (SUCRA = 79.6%) > Astragalus membranaceus injection + conventional therapy (SUCRA = 73.8%) > Chuanxiong Rhizoma injection + conventional therapy (SUCRA = 70.1%) > Danhong injection + conventional therapy (SUCRA = 38.9%) > Shenmai injection + conventional therapy (SUCRA = 34.2%) > Shenxiong injection + conventional therapy (SUCRA = 31.0%) > Guanxin Ning injection + conventional therapy (SUCRA = 22.9%) > Shenfu Injection + conventional therapy(SUCRA = 2.1%); (for details, see Fig. 5G and Table 9).

**Table 9:** Results of network meta-analysis of LVESV (MD, [95% CI]).

Interventions	CT	CXQ + CT	DH + CT	DSDFSY + CT	GXN + CT	HQ + CT	SF + CT	SM + CT	SX + CT
CT	0	/	/	/	/	/	/	/	/
CXQ + CT	3.60 (−3.57, 10.77)	0	/	/	/	/	/	/	/
DH + CT	9.00 (6.71, 11.29)	5.40 (−2.13, 12.93)	0	/	/	/	/	/	/
DSDFSY + CT	1.99 (−0.38, 4.36)	−1.61 (−9.17, 5.95)	−7.01 (−10.30, −3.71)	0	/	/	/	/	/
GXN + CT	11.23 (5.71, 16.75)	7.63 (−1.42, 16.68)	2.23 (−3.74, 8.21)	9.24 (3.23, 15.25)	0	/	/	/	/
HQ + CT	2.68 (1.38, 3.99)	−0.92 (−8.21, 6.38)	−6.31 (−8.95, −3.68)	0.69 (−2.01, 3.40)	−8.55 (−14.22, −2.87)	0	/	/	/
SF + CT	14.90 (12.62, 17.18)	11.30 (3.77, 18.83)	5.90 (2.67, 9.14)	12.91 (9.62, 16.20)	3.67 (−2.30, 9.65)	12.22 (9.59, 14.85)	0	/	/
SM + CT	9.50 (3.48, 15.52)	5.90 (−3.47, 15.27)	0.50 (−5.94, 6.95)	7.51 (1.04, 13.98)	−1.73 (−9.90, 6.44)	6.82 (0.65, 12.98)	−5.40 (−11.84, 1.04)	0	/
SX + CT	9.79 (8.21, 11.37)	6.19 (−1.15, 13.54)	0.80 (−1.99, 3.58)	7.80 (4.95, 10.65)	−1.44 (−7.18, 4.31)	7.11 (5.06, 9.16)	−5.11 (−7.88, −2.33)	0.29 (−5.93, 6.52)	0

CI = confidence interval, CT = conventional therapy, CXQ = Chuanxiong Rhizoma, DH = Dan Hong, DSDFSY = Salvianolate, GXN = Guanxin Ning, HQ = Astragalus membranaceus, LVESV = left ventricular end-systolic volume, MD = mean difference, SF = Shenfu, SM = Shenmai, SX = Shenxiong.

### 3.12. Publishing biased evaluations

**Figure 6.**
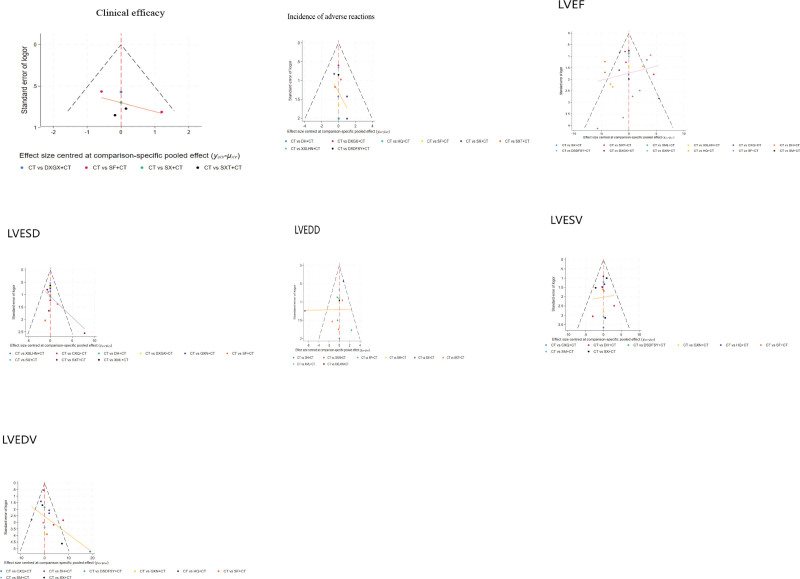
Corrected funnel plot results for each outcome indicator. (This article is original and no conflict of interest exists).

Corrected funnel plots were drawn for the 7 outcome indicators, in which the clinical efficacy indicators were included in fewer literatures, and the test efficacy of the funnel plot results was insufficient and the reference significance was low, so they were only presented as results. The results of the remaining indicators showed that: LVEF, LVEDD, LVESV had good symmetry and a gentle regression line, suggesting a small publication bias; while the incidence of adverse reactions, LVESD, LVEDV had poor symmetry and a steeper regression line, suggesting a large publication bias or a small-sample effect. (See Fig. 6 for details).

### 3.13. Sensitivity analysis

Taking the clinical efficacy rate index as an example, sensitivity analysis was carried out by the exclusion method one by one. The results showed that the effect estimates of individual studies did not change significantly, suggesting that the overall analysis results were robust to the exclusion of individual studies. (See Table 10 for details).

**Table 10:** Sensitivity analysis of clinical efficacy rates included in the literature.

Included literature	OR (95% CI)
Yan Hua, 2017^[10]^	2.75 (1.56–4.85)
Bao Bohang, 2020^[11]^	4.21 (2.25–7.88)
Luo Qi, 2020^[13]^	2.92 (1.64–5.20)
Liu Boxin, 2024^[21]^	2.92 (1.60–5.34)
Liu Chunxiao, 2016^[26]^	3.04 (1.74–5.32)
Li Bo, 2018^[27]^	2.90 (1.65–5.11)

## 4. Discussion

A total of 12 different herbal injections were included in this study, and the outcome indicators of clinical efficacy, incidence of adverse reactions, LVEF, LVEDD, LVESD, LVEDV, LVESV were selected according to the 2020 Expert Consensus on Prevention and Treatment of Ventricular Remodeling after AMI^[32]^ and Guidelines for Clinical Application of Echocardiographic Assessment of Cardiac Systolic and Diastolic Function^[33]^ as the Net Meta system to evaluate the efficacy and safety of ventricular remodeling after AMI, in order to reflect the changes of each treatment effect as objectively as possible. The clinical efficacy, as one of the key indexes of evaluation, was chosen to analyze LVEF, LVEDD, LVESD, LVEDV, LVESV indexes as the basis of evaluation of its efficacy due to the small number of literature included and the different criteria for judging the efficacy of some of them, which led to the lack of referentiality and statistical significance. For the indicator of the incidence rate of adverse reactions, the differences were not statistically significant (*P* >.05) due to the small difference in the actual number of occurrences between the experimental groups and the control group, the relatively small-sample size included, and the inconsistency in the criteria for the selection of the adverse reaction events, etc. Therefore, it is necessary to conduct further research to prove whether the incidence of the adverse reactions of the various traditional Chinese medicines injections assisting the conventional treatment is significantly reduced as compared with the conventional treatment or whether the incidence of the adverse reactions needs to be increased by more Data and sample size validation of complete and uniform standards. The final results showed that: In terms of clinical efficacy, the efficacy of Shuxuetong injection in assisting conventional treatment may be the best, followed by Shenxiong injection; according to the study,^[34]^ Shuxuetong injection possesses the efficacy of traditional Chinese medicines in activating blood circulation and removing blood stasis, and promoting the circulation of meridians and collaterals, and also possesses the functions of endothelial function,^[35]^ anti-inflammation,^[36]^ and inhibition of ventricular remodeling^[28]^ in treating AMIs; The occurrence rates of adverse reactions In terms of enhancement of LVEF value, Salvianolate injection assisting conventional treatment occurred relatively least adverse reactions and was the safest, followed by shenxiong injection; studies have shown that Salvianolate injection has the efficacy of activating blood circulation and removing blood stasis, nourishing the blood and tranquilizing the mind,^[37]^ and it also protects against ischemia-reperfusion injury and improves microcirculation, etc.^[38]^ In terms of enhancement of LVEF value, Guanxin Ning injection assisting conventional treatment may be the best, followed by Danhong injection. followed by Danhong injection; Guanxin Ning injection can nourish blood and activate blood, regulate qi and stop pain,^[39]^ increase myocardial oxygen supply and improve cardiac function, etc.^[40]^ In terms of lowering the LVEDD value, Xinmailong injection assisting conventional treatment may be the best, followed by Xingxiong sodium chloride injection; Xinmailong injection can warm yang and promote water retention, benefit qi and activate blood, as well as vasodilatation, improve endothelial function, inhibit ventricular remodeling, and other^[41]^ modern medical effects. In terms of lowering LVESD values, Shenxiong injection adjunctive conventional treatment may be the best, followed by Xinmailong injection; studies have shown^[42]^ that Shenxiong injection has the effects of activating blood circulation, removing blood stasis, and vasodilating blood vessels. In terms of lowering LVEDV values, Chuanxiong Rhizoma injection adjunctive conventional treatment may be the best, followed by Shenfu injection; and Chuanxiong Rhizoma injection has the effects of activating blood circulation and removing blood stasis, protecting the heart, improving microcirculation, and inhibiting apoptosis of cardiomyocytes.^[43]^ In terms of lowering LVESV value, Shenfu injection may be the best in assisting conventional treatment, followed by Guanxin Ning injection; relevant studies have confirmed^[44]^ that Shenfu injection usually has the effects of benefiting qi to consolidate and remove blood stasis and returning yang to save reversal of the heart, and it also improves the cardiac function and reduces the cardiac load. The above results can provide a basis for the clinical drug selection program, which has certain guiding significance. This study, as a reticulated Meta-analysis, compares the efficacy and safety of different TCM injections assisting conventional therapy in the treatment of ventricular remodeling after AMI, which is more systematic and comprehensive than the traditional Meta-analysis, and at the same time there are the following shortcomings and limitations: the quality of the included literatures was low, and most of them did not describe “concealed allocation scheme,” “blinding method,” “other sources of bias,” etc. Most of the sample sizes were relatively small, which may cause a certain risk of bias, which may affect the reliability of the final analysis results. Lack of head-to-head studies between the interventions of various Chinese medicine injections to assist conventional treatment that did not form a ring, resulting in a lack of directly comparable results; Among different Chinese medicine injections, some of the injections were included in too little literature, and small-sample effects may occur; The included literature was basically all in Chinese, and the research subjects were all in China, which may not be recognized enough as research abroad; the research subjects of each literature may have a part of the degree of illness and the difference in the instrumentation of the measurement indexes, which may also cause an impact on the impact of the results; included in the study of the clinical efficacy of the assessment of less, and the lack of a unified standardized standards; in view of this, the actual clinical selection of drugs, or should be in the specific circumstances, guidelines, and other prerequisites objectively combined with the results of this study.

## 5. Summary

In conclusion, the adjuvant treatment of Chinese medicine injection on the basis of conventional treatment can safely and effectively improve the degree of ventricular remodeling after AMI and enhance the cardiac function, quality of life and prognosis of patients to different degrees, which provides a broader idea and sufficient basis for the treatment of patients with ventricular remodeling after AMI with the combination of traditional Chinese medicine and Western medicine. Meanwhile, the conclusions of this study are not completely reliable and still need to be evaluated and verified by incorporating future RCTs such as large sample size, high quality, and head-to-head studies, so as to make TCM injection better and more rationally serve in clinical work.

## Author contributions

**Data curation:** Huan Wu.

**Writing – original draft:** Jianlong Xiong.

**Writing – review & editing:** Tao Xu.
